# Spatial and Temporal Persistence of Fluorescent *Lactiplantibacillus plantarum* RS-09 in Intestinal Tract

**DOI:** 10.3389/fmicb.2022.843650

**Published:** 2022-03-30

**Authors:** Xiaoyu Zhao, Chenpei Zhao, Leining Yang, Linlin Jiang, Jianlong Zhang, Xin Yu, Guozhong Chen, Hongwei Zhu, Wenli Tang, Youzhi Li, Maolian Wei, Xingxiao Zhang, Hong Jia

**Affiliations:** ^1^School of Life Sciences, Ludong University, Yantai, China; ^2^Department of Prosthodontics, Yantai Stomatological Hospital Affiliated to Binzhou Medical University, Yantai, China; ^3^Shandong Aquaculture Environmental Control Engineering Laboratory, Yantai, China; ^4^Shandong Provincial Key Laboratory of Quality Safety Monitoring and Risk Assessment for Animal Products, Institute of Veterinary Drug Quality Inspection of Shandong Province, Jinan, China; ^5^Institute of Animal Sciences, Chinese Academy of Agricultural Sciences, Beijing, China

**Keywords:** *Lactiplantibacillus plantarum*, adherence, persistence, CFDA/SE, colonization

## Abstract

The beneficial effects of the probiotic strain *Lactiplantibacillus plantarum* (formerly *Lactobacillus plantarum*) are based on its adherence and colonization ability in the gut. However, little is known about the migration and long-term gut colonization of the strain. This study evaluated the gut colonization modes of *Lactiplantibacillus plantarum* RS-09 to identify the strain with long-term gut colonization potential. We established CFDA/SE-labeled RS-09 to study the temporal and spatial distribution of RS-09 in the intestine as well as to analyze its persistence in different parts of the intestine by flow cytometry. This study has shown that the RS-09 strain maintains strong adhesion abilities under acid (pH 2.5) and base (pH 8.5) conditions. In addition, CFDA/SE can be used as an indicator for the labeling of *L. plantarum* RS-09 in the intestinal tract *in vivo*. We established a growth kinetics model of RS-09 to elucidate its persistence in the intestine. *In vivo* persistence experiments showed that the persistence rate of RS-09 was the highest in the cecum (69.5%) and the lowest in the duodenum (12.8%) at 96 h. After 20 days, RS-09 was predominantly localized in the cecum and colon steadily. These studies provide new insights into the long-term persistence of *L. plantarum* in the gastrointestinal tract. The CFDA/SE label system may be used to study the *in vivo* colonization dynamics of other probiotic strains.

## Introduction

*Lactobacillus* species are Gram-positive, homofermentative, thermophilic and non-spore-forming rods (Zheng et al., 2020). It is a beneficial microorganism commonly found in the gastrointestinal tract of humans and animals (Seddik et al., 2017). *Lactiplantibacillus plantarum*, previously named “*Lactobacillus plantarum*,” is one of the most widely distributed bacterium (Fidanza et al., 2021). It is acid resistant and is commonly found in fermented plant foods such as kimchi, silage, fruits and beverages (Barache et al., 2020). It has important physiological functions. Studies have shown that *L. plantarum* is a dominant bacterium in the intestine of humans and animals and has strong antibacterial activity against pathogenic microorganisms (Zhu et al., 2014; Li et al., 2016), as well as good tolerance to various stresses, including those present in the gastrointestinal tract. In view of the safety of *L. plantarum* and its beneficial effects on human health, a large number of studies have been conducted to isolate and screen new strains with probiotic characteristics (Goel et al., 2020; Garcia-Gonzalez et al., 2021) and the ability to sustain processing conditions for culture production (Sauvageau et al., 2012).

According to the Food and Agriculture Organization of the United Nations/World Health Organization (FAO/WHO) regulations (2002), probiotics must have a high tolerance to the gastrointestinal (GIT) conditions as well as high adherence in order to serve as a probiotic and maintain the balance of the intestinal microbiota (Hardy et al., 2013; Monteagudo-Mera et al., 2019). Studies have shown that gastrointestinal conditions (pH, bile, digestive enzymes) and the acid and bile salt tolerance of bacteria have important impacts on the adhesion ability of probiotics (Damaceno et al., 2021; Pumriw et al., 2021; Tarrah et al., 2021). To ensure that *L. plantarum* is able to survive after passage through the gastrointestinal tract, evaluating the effect of acid-base stress on this organism has important significance for studying its adhesion ability.

The ability to adhere to the intestinal mucosa is another important criterion for screening probiotics (Chua et al., 2020). Adhesion can prolong the amount of time that probiotics are present in the intestines and allows for close contact between probiotics and intestinal epithelial cells. Different methods and models have been used to assess the adhesion of probiotics. HT-29 cell lines are the main cell models for adhesion ability assessments, both within and outside of China because these cell lines consist of mature intestinal epithelial cells (Trukhachev et al., 2021). However, bacterial persistence is easily antagonized by other strains in the intestinal microbiota. It is not sufficient to test probiotic adhesion abilities through *in vitro* experiments alone and more convincing evidence is needed to confirm *in vitro* results (Papadimitriou et al., 2015). Therefore, it is important to study the dynamic distribution and persistence patterns of lactobacilli in different segments of the gastrointestinal tract.

In recent years, flow cytometry (FCM) has been widely used for real-time monitoring and quantitative analysis of single cells due to its advantages of high speed, high precision and good accuracy. This technique lays the foundation for studying whether probiotics can adhere to and colonize host cells. For example, the adherence of flourescein isothiocyanate (FITC)-labeled *L. plantarum* can be clearly observed by fluorescence microscopy, which allows for a quantitative approach for studying adhesion to intestinal epithelial cells. However, FITC has disadvantages, including relatively high levels of photobleaching, high pH sensitivity and a broad spectrum, which make quantitative measurements of this fluorophore inaccurate and thereby reduces the effectiveness of detection (Xing et al., 2018). Carboxyfluorescein diacetate succinimidyl ester (CFDA/SE) is a lipophilic molecule that is minimally fluorescent until it enters cells by passive diffusion and esterases cleave the acetyl groups to form carboxyfluorescein succinimidyl ester (CFSE), which irreversibly reacts with the lysine residues of intracellular proteins to produce fluorescence (Cong et al., 2008). When a CFDA/SE-labeled cell divides, its progeny is endowed with half the number of carboxyfluorescein-tagged molecules and thus each cell division can be assessed by measuring the fluorescence *via* Flow cytometry. In addition, CFDA/SE efficiently stained bacteria without causing undesirable effects on cell adhesion or viability (Fuller et al., 2000). CFDA/SE has been widely used for bacterial labeling and more than 95% of CFDA/SE-stained bacterial strain incubated in artificial groundwater remained fluorescent for at least 28 days as determined by epifluorescent microscopy and flow cytometry (Fuller et al., 2000). However, no reports are available on the dynamic monitoring of CFDA/SE-labeled *L. plantarum* in the intestinal tract of animals.

Plant origin lactic acid bacteria (POLAB) have received increasing attention due to the ability to cope with multiple stresses (Lim et al., 2018). POLAB can often produce natural antibacterial and physiologically active substances. In this study, we isolated and purified *L. plantarum* strains from apple fermented juice. In this work, a reliable, rapid, and simple quantitative method was established using FCM to study the influence of *L. plantarum* adherence and persistence on intestinal epithelial cells. CFDA/SE fluorescent labeling was used for long-term dynamic monitoring of the distribution and persistence of *L. plantarum* in the intestine. Thereby, we can understand the adhesion processes of lactic acid bacteria *in vivo* and by fitting mathematical mode, we can obtain the persistence patterns of lactic acid bacteria in the intestine.

## Materials and Methods

### Bacterial Strains and Culture Conditions

*Lactiplantibacillus plantarum* RS-09 is a lactic acid bacterium successfully isolated from fermented apple and stored in our laboratory. The strain is stored in the China General Microbiological Culture Collection Center (CGMCC No. 17118). The glycerol-preserved RS-09 strain was inoculated into freshly prepared MRS broth (Solarbio, Beijing, China) and incubated under anaerobic conditions at anaerobe container system at 37°C for 24 h to obtain the bacterial suspension.

### Strain Identification

A fully automated microbial identification system (VITEK 2 Compact, BioMérieux, Lyon, France) was used to analyze the biochemical reactions of the strain. The VITEK^®^ 2 Anaerobic and Corynebacterial identification card was used to identify the isolated strains (Pence and Liesman, 2020). The identification results were compared to the data in the BioMérieux database.

We used the universal primers, 27F (5′-AGAGTTTGATCCTGGCTC AG-3′) and 1492R (5′-TACGGYTACCTTGTTACGACTT-3′), for the amplification of 16S rDNA using the Rapid PCR kit (Qiagen, Germantown, MD, United States). The 16S rDNA was sent to Invitrogen for sequencing. BLAST was used to analyze the sequences in comparison to those in the GenBank. The phylogenetic tree was built *via* the Neighbor-joining method using the MEGA 7 software.

### Cells and Cell Culture

An HT-29 cell lines was cultured in Dulbecco’s modified Eagle’s medium (DMEM, Hyclone, NY, United States) supplemented with fetal bovine serum (Gibco, Grand Island, NY, United States) and incubated at 37°C in a water-jacketed incubator with 5% carbon dioxide. The cells were harvested by trypsinisation (0.25%, Sigma, St. Louis, MO, United States), washed with sterile phosphate buffer saline (PBS, beyotime, Shanghai, China), and counted using a hemocytometer. Cells were fed with a change of medium every 2 days. When reached about 80 per cent confluency, cells were harvested for the next experiment.

### Acid and Bile Salt Tolerance

For testing the acid tolerance, the previously described method was followed with some modifications (Hussein et al., 2020). RS-09 was cultured in MRS broth at 37°C for 24 h. After incubation, 200 μL of the bacterial suspension was inoculated into 2 mL MRS broth, supplemented with HCl to mimic the gastric acidic conditions. The pH of the broth was adjusted to 1.5, 2.5, 3.5, 4.5 and 5.5, respectively, with 1 mol/L HCl. The bacterial solution was anaerobically cultured at 37°C for 2 h, 500 μL bacterial suspension was taken immediately before and after the incubation, and 10-fold serially diluted before spread-plating on MRS agar. After incubation at 37°C for 48 h, the colonies were counted. MRS medium (pH = 6.5) was used in the control group, and the survival rate was calculated using the following formula: survival rate (%) = (viable cell count in the treated group/viable cell count in the control group) × 100.

The bile salt tolerance of the strain was determined according to the method reported by Prins et al. (2010). In brief, the broth was supplemented with bile salts (Solarbio, Beijing, China) to final concentrations of 0.3, 0.5, 1.0, 1.5, and 2.0% (w/v), respectively. Cells grown in MRS broth without bile salt was used as control. The bacterial suspension was cultured for 2 h and inoculated onto MRS agar for colony counting. The survival rate was calculated using the following formula: survival rate (%) = (viable cell count in the treated group/viable cell count in the control group) × 100.

### Scanning Electron Microscope Analysis

*Lactiplantibacillus plantarum* RS-09 was treated at different pH levels for 24 h, and the cells were collected by centrifugation (4,000 *g*, 10 min). For SEM, the bacteria were fixed with 2.5% glutaraldehyde for 2 h, and washed with phosphate buffer solution (PBS, pH 7.4). The samples were dehydrated with a series of density ethanol solutions (30, 50, 70, 80, 90, and 100%). Finally, the samples were replaced in tertiary-butanol alcohol at concentrations of 25, 50, and 75%. Samples were freeze dried for 24 h by Vacuum Freeze Dryer (Christ, Alpha 1–4 LDplus, Germany). Finally, the samples were covered with gold-plated membrane and observed with a Hitachi S-3400N (Hitachi, Tokyo, Japan).

### CFDA/SE Staining of Cultured Bacteria

*Lactiplantibacillus plantarum* RS-09 was inoculated into freshly prepared MRS medium and anaerobically cultured to log phase at 37°C. The medium was then centrifuged (5,000 *g*, 10 min, 4°C) and the pellet was washed twice with pre-cooled phosphate-buffered saline (PBS) (pH = 7.2) and resuspended in PBS containing 20 μmol/L CFDA/SE (Solarbio, Beijing, China). After incubation at 37°C for 30 min in the dark, the samples were washed 5 times in PBS to remove unbound CFDA/SE and the bacteria were resuspended in MRS medium at a concentration of 10^8^ CFU/mL, followed by anaerobic culture at 37°C for 0, 24, 48 and 72 h. The bacteria were collected and observed under a fluorescence microscope with excitation at 488 nm and analyze the positive rate by FCM.

### Adhesion Assay

Human colon adenocarcinoma HT-29 cells in a monolayer were washed with PBS, and seeded separately in each well of 6-well plates containing cover slips. A monolayer of HT-29 cells (1 × 10^5^ each well) was used for adhesion assays. Labeled or non-labeled *L. plantarum* strains cell pellet (10^8^ CFU) collected *via* centrifugation (5,000 *g*, 10 min, 4°C) was then suspended in a DMEM medium to each well of a 6-well plate containing HT-29 cells and incubated at 37°C with 5% CO_2_ for 2 h. The 6-well plate was washed five times with PBS to remove non-adherent bacteria. For microscopic examination of adhered non-labeled *L. plantarum*, the bacteria were treated with Gram staining. The plate was air dried and examined under the inverted microscope (Olympus BX41, Japan, Olympus Corporation). A fluorescent microscope was used to observe the labeled *L. plantarum* adhering to the HT-29 cells. The adhesion ratio of *L. plantarum* on HT-29 cells was calculated by comparing the viable count using flow cytometry before and after adhesion. The following formula: survival rate (%) = (viable cell count in the treated group/viable cell count in the control group) × 100. Each assay was conducted at the same time in three independent experiments.

### Intestinal Distribution of *L. plantarum* RS-09 in Mouse

All mouse experimental procedures used in this study were approved by the Laboratory Animal Management Committee of Ludong University. The animal model was established to monitor the temporal and spatial distribution of A fluorescently-labeled *L. plantarum* RS-09 in the gastrointestinal tract *in vivo*. A total of 180 C57B/L6 male mice aged 4–5 weeks were randomly divided into two experiments, with 90 mice in each experiments. One was used for FCM analysis and the other was used to obtain frozen tissue sections for observation.

The *L. plantarum* RS-09 strain was collected during log-phase growth, labeled with CFDA/SE and resuspended to 1 × 10^10^ CFU/mL in PBS. Then, 500 μL of cell suspension was administered to each mouse in the experimental group, while 500 μL of sterile PBS was administered to each mouse in the control group. At different time points, the contents from different parts of the intestine were collected and flow cytometric analysis was performed to determine the rate of CFDA/SE-labeled *L. plantarum*. Statistical analysis was performed using logistic regression to determine the relationship between variables (CFDA/SE and microbial count). MATLAB (Release 2018b, The MathWorks Inc., Natick, MA, United States) was used for data analysis to establish a growth kinetics model of CFDA/SE-labeled *L. plantarum* RS-09 persistence in the intestine. The model of bacterial count was fitted by the Fourier series function. The model equations are:


$$f\,\left(x\right)=a_{0}+\sum_{i=1}^{n}\left(a_{i}\,\cos\left(\mathrm{iwx}\right)+b_{i}\,\sin\left(\mathrm{iwx}\right)\right)$$

where a_0_ models a constant (intercept) term in the data and is associated with the *i* = 0 cosine term, w is the fundamental frequency of the signal, x is in hours from 0 to 480, n is the number of terms in the series. The values in the ileum were fitted using the sixth order Fourier series, in the cecum using the seventh order Fourier series and in the colon using the fourth order Fourier series.

### Flow Cytometric Analysis

All experiments were performed using a FACS Calibur flow cytometer (Becton Dickinson, San Jose, CA, United States) equipped with an air-cooled 15 mW argon ion laser, emitting at a fixed wavelength of 488 nm. Fluorescent filters and detectors were all standard with green fluorescence collected in the FL1 channel (530 F 30 nm). All parameters were collected as logarithmic signals. Data were analyzed using FlowJo Software (FlowJo, LLC, Ashland, OR, United States).

### Fluorescent Detection of *L. plantarum* RS-09 in Histological Slices

Samples of the ileum, cecum, and colon were harvested at different time points for frozen section preparation and observation. The tissue samples were fixed in a 4% formalin solution overnight. The formalin-fixed tissue samples were dehydrated with 10, 20, and 30% sucrose solutions for 30 min each. The tissue samples were embedded with a frozen embedding agent (OCT compound; Sakura, Japan) and frozen at −20°C for at least 30 min. Then, the embedded samples were held for 10 min in a precooled freezing microtome (Leica, Wetzlar, Germany), followed by sectioning. The frozen sections were observed under the fluorescence microscope.

### Statistics

Statistical analysis was performed using SPSS V17.0 (SPSS Inc., Chicago, IL, United States) and figures were generated using GraphPad Prism version 5.0 (GraphPad Software Inc., San Diego, CA, United States). The data were expressed as the means ± SDs. The mean values obtained from the experiments were compared using Student’s test or one-way ANOVA. A *P*-value less than 0.05 was considered significant.

## Results

### Strain Identification

A fully automated, microbial identification system was used for the identification of the isolated strain. The accuracy of identification by VITEK was 91%. The results showed that the isolated strain was *L. plantarum* (Table 1). This strain can utilize a variety of sugars and sugar alcohols, including galactose, glucose and mannitol. However, this strain cannot utilize urease, D-xylose, or L-arabinose.

**TABLE 1:** Analysis of *L. plantarum* RS-09 using the automated microbial identification system.

Name	Abbreviation	Result
D-Galactose	dGAL	+
Leucine Arylamidase	LeuA	+
ELLMAN	ELLM	−
Phenylalanine Arylamidase	PheA	+
L-proline Arylamidase	ProA	−
L-Pyrrolydonyl-Arylamidase	PyrA	−
D-cellobiose	dCEL	+
Tyrosine Arylamidase	TyrA	−
Alanine-phenylalanine-proline Arylamidase	APPA	−
D-Glucose	dGLU	+
D-Mannose	dMNE	+
D-Maltose	dMAL	+
Saccharose	SAC	+
Arbutin	ARB	+
*N*-acetyl-D-glucosamine	NAG	+
5-Bromo -4-chloro -3-indoxyl-beta-glycoside	BGLUi	+
Urease	URE	−
β5-Bromo -4-chloro -3-indoxyl-beta-glucuronide	BGURi	−
Beta-galactopyr anosidase indoxyl	BGALi	+
Aerobic	AERO	−
Alpha-Arabinosidase	AARA	−
5-Bromo -4-chloro -3-indoxyl-alpha-galactoside	AGALi	−
Beta-mannosidase	BMAN	−
Arginine GP	ARG	−
Pyruvate	PVATE	−
Maltotriose	MTE	+
Esculin hydrolyze	ESC	+
B eta-D-Fucosidase	BdFUC	−
5-Bromine -4-chloride -3-hydroxyapatite-β-*N*-acetyl-glucosamine	BNAGi	−
5-Bromo -4-chloro -3-indoxyl-alpha-mannoside	AMANi	−
Alpha-Fucoidansidase	AIFUC	−
Phospatase	PHOS	−
L-arabinose	LARA	−
D-ribose 2	dRIB2	+
phenylphosphonate	OPS	−
Alpha-L-Arabinofur anosidase	AARAF	−
D-xylose	dXYL	−
Gram-positive	GRAM	+
morphology	MORPH	+

*“+”: Physiological and biochemical tests positive.*

*“−”: Physiological and biochemical tests negative.*

**FIGURE 1:**
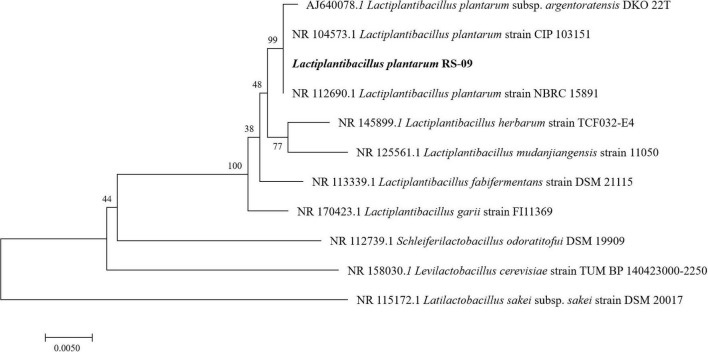
Phylogenetic trees based on 16S rDNA gene sequences showing the relationship between *Lactiplantibacillus plantarum* RS-09 and closely related species. Neighbor-joining dendrograms were generated with bootstrap trials of 500.

The sequencing results demonstrated that the 16S rDNA contained 1,385 base pairs, which were compared with the sequences in GenBank to draw a phylogenetic tree, as shown in Figure 1. Phylogenetic analysis showed 99% identity to the *Lactiplantibacillus plantarum* strain. The strain was preliminarily named as *Lactiplantibacillus plantarum* RS-09. Figure 1 demonstrated the relationship of RS-09 with other closely related *L. plantarum* using 16S rDNA gene sequences.

### Acid and Bile Tolerances of *L. plantarum* RS-09

Resistance to gastric pH and bile are key features for bacteria to be able to survive in the gastrointestinal tract (GIT) and are some of the important selection criteria for probiotic. We tested the acid resistance of *L. plantarum* RS-09 at pH 1.5, 2.5, 3.5, 4.5 and 5.5. The results demonstrated that RS-09 significantly survived in the harsh pH condition (pH 1.5; 87.91%). After incubation at pH 2.5, 3.5, 4.5 and 5.5, the survival rates were greater than 90%, but without statistical difference (*P* > 0.05) (Table 2).

**TABLE 2:** Acid tolerance of *Lactiplantibacillus plantarum* RS-09 at different pH conditions for 2 h (*n* = 3, mean ± SD).

pH	Viable count (1 × 10^6^ CFU/mL)^a^		Survival rate (%)^b^
	0h	2h	
1.5	5.10 ± 0.50	4.48 ± 0.38	87.91
2.5	5.58 ± 0.65	5.37 ± 0.54	96.12
3.5	6.55 ± 1.55	6.40 ± 1.00	97.71
4.5	7.01 ± 0.18	7.00 ± 0.35	98.82
5.5	8.10 ± 0.40	8.22 ± 0.42	101.44

*^a^Values are means ± standard deviation of three independent experiments.*

*^b^Acid resistant strains with mean survival rates.*

A 0.3–2.0% bile salt concentration was chosen for simulation of a bile salt challenge. At 37°C, the survival rate of *L. plantarum* RS-09 in 0.3% bile salt was 71.43%. When the bile salt concentration in the MRS media was increased to 1.5 or 2.0%, the survival rates were 68.95 and 62.76%, but without statistical difference (*P* > 0.05) (Table 3).

**TABLE 3:** Alkali resistance of *L. plantarum* RS-09 at different bile salt concentrations for 2 h (*n* = 3, mean ± SD).

Bile salt (%)	Viable count (1 × 10^6^ CFU/mL)*^a^*		Survival rate (%)*^b^*
	0h	2h	
0.3	7.82 ± 0.34	5.58 ± 0.53	71.43
0.5	7.57 ± 0.10	5.33 ± 0.28	70.48
1	7.38 ± 0.67	5.20 ± 0.28	70.43
1.5	7.30 ± 0.58	5.03 ± 1.27	68.95
2	7.12 ± 0.85	4.47 ± 0.73	62.76

*^a^Values are means ± standard deviation of three independent experiments.*

*^b^Acid resistant strains with mean survival rates.*

### Scanning Electron Microscope Analysis of *L. plantarum* RS-09

**FIGURE 2:**
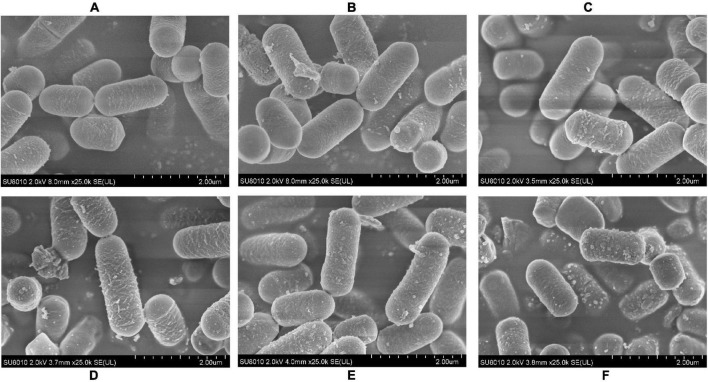
SEM images of *L. plantarum* RS-09 from control and the acid groups. **(A)** Control group; **(B)** pH 5.5 group; **(C)** pH 4.5 group; **(D)** pH 3.5 group; **(E)** pH 2.5 group; **(F)** pH 1.5 group.

**FIGURE 3:**
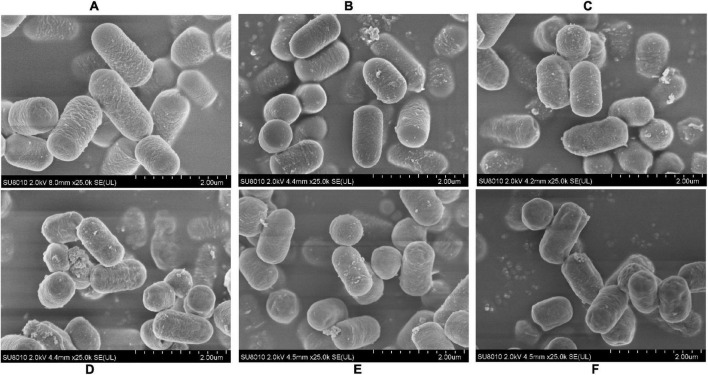
SEM images of *L. plantarum* RS-09 from control and bile salt groups. **(A)** Control group; **(B)** 0.3% bile salt; **(C)** 0.5% bile salt; **(D)** 1% bile salt; **(E)** 1.5% bile salt; **(F)** 2% bile salt.

To determine if the pH stress did some damage to the cell membranes and affected the adhesive ability of the *L. plantarum* RS-09, we used SEM to observe the morphological changes of *L. plantarum* RS-09 cells. As shown in Figure 2, *L. plantarum* RS-09 is shaped as an elongated rod under acidic conditions but appears as a short rod with thick pili under basic condition. Under strong basic conditions, the structures of some *L. plantarum* RS-09 cells were fold, with cells wall collapsed (Figure 3). The acid resistance ability was stronger compared with the strains in the alkali stress conditions.

### CFDA/SE Can Efficiently Label *L. plantarum* RS-09

**FIGURE 4:**
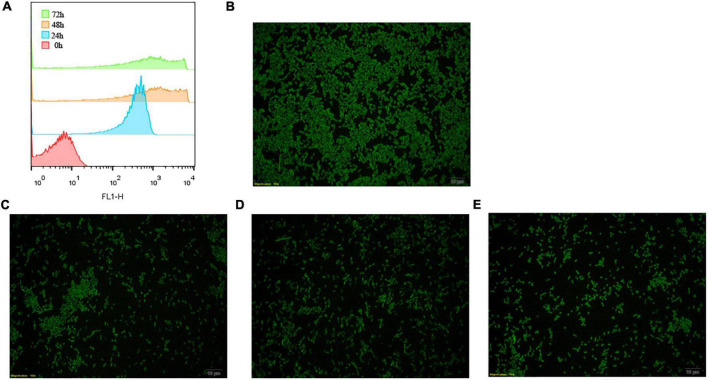
Fluorescent detection of labeled *L. plantarum* RS-09. **(A)** Flow cytometry results for the detection of fluorescently labeled RS-09; **(B–E)** CFDA/SE labeled *L. plantarum* cultured in MRS for 0, 24, 48, and 72 h investigated under fluorescence microscopy.

The CFDA-SE label was stable and has been described for various cells. To determine how about the CFDA-SE labeling stability, cells were cultured at low density and at indicated time points, analyzed by flow cytometry and fluorescence microscopy. With each cell division, the label was diluted, as it was divided between daughter cells. Initially, 99% of the cells were labeled (Figure 4). The intensity of CFDA-SE fluorescence was still high in each cell during 72 h. These results are consistent with those obtained by fluorescence microscopy, indicating that CFDA/SE can effectively label *L. plantarum* for long-term monitoring of these strains.

### *Lactiplantibacillus plantarum* RS-09 Adhesion Ability

**FIGURE 5:**
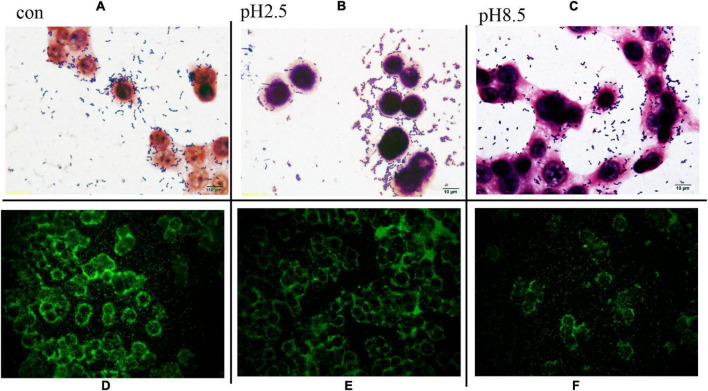
*L. plantarum* RS-09 adhesion assay on HT-29 cells under different pH stress. **(A–C)** Adhesion observation by Gram stain. Scale bars, 10 μm (insert, 1,000×). **(D–F)** Fluorescence microscopic to analyze *L. plantarum* RS-09 adhesion on HT-29 cells. In each case, same number of *Lactobacillus* cells (1 × 10^8^ CFU/mL) were applied and treated equally. Scale bars, 20 μm (original magnification, 400×).

One of the important properties of lactobacilli is their ability to adhere to the target sites for their colonization in the gut for expressing optimal functionality. The ability of *L. plantarum* RS-09 to adhere to HT-29 cells is shown in Figure 5. Gram staining showed that *L. plantarum* RS-09 was highly adherent to the HT-29 cells (Figure 5). To further observe the ability of *L. plantarum* RS-09 to adhere to cells, the CFDA/SE-labeled strain was incubated with the cells for 2 h, followed by observation by fluorescence microscopy (Figure 5) and FCM analysis for bacterial count. In our study, adhesion of *L. plantarum* RS-09 on HT-29 cells was quantified (Table 4). The results showed that the adhesion rate of RS-09 to HT-29 cells was 7.85%, respectively. Medium with different pH conditions was used to observe the ability of the RS-09 strain to adhere to intestinal epithelial cells. The results showed that under strong acid and weak base conditions, the RS-09 stain still had a high rate of adhesion, where the adhesion rates were 7.73% at pH 2.5 and 5.01% at pH 8.5. The adhesion ability under acid resistance was stronger compared with the strains in the alkali stress conditions.

**TABLE 4:** Adhesion rate of *L. plantarum* RS-09 at different pH conditions (*n* = 3, mean ± SD).

pH	Viable count (1 × 10^6^ cfu/mL)*^a^*		Adhesion rate (%)
	0h	2h	
control	100	7.85 ± 0.16	7.85 ± 0.16
1.5	100	6.85 ± 0.35	6.85 ± 0.35
2.5	100	7.73 ± 1.11	7.73 ± 1.11
3.5	100	7.70 ± 0.40	7.70 ± 0.40
4.5	100	7.71 ± 0.08	7.71 ± 0.08
5.5	100	6.98 ± 0.14	6.98 ± 0.14
8.5	100	5.07 ± 0.51	5.07 ± 0.51

*^a^Values are means ± standard deviation of three independent experiments.*

### Using CFDA/SE-Labeled *L. plantarum* RS-09 to Study Persistence Patterns

**FIGURE 6:**
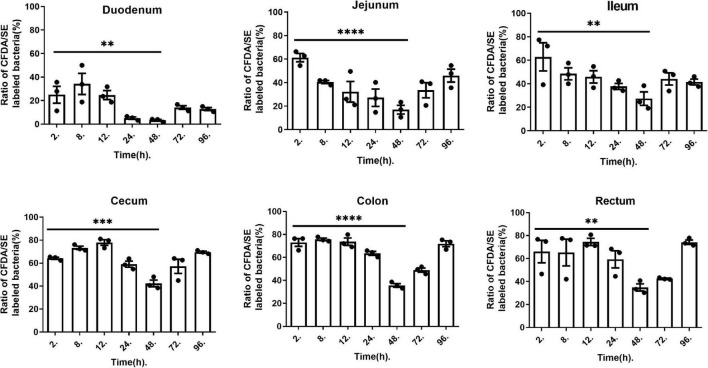
*L. plantarum* RS-09 fluorescence at different time points and measured in different organs. Data are presented as mean ± SEM. One-way ANOVA in SPSS Statistic. **p < 0.01, ***p < 0.001, and *⁣*⁣**p < 0.0001.

To better confirm the adherence and persistence process of *L. plantarum* RS-09 in the gastrointestinal tract, CFDA/SE-labeled strains can be used for long-term monitoring due to their stable fluorescence. The intestinal contents were analyzed by FCM at 2, 8, 12, 24, 48, 72 and 96 h. The concentrations of *L. plantarum* in different segments of the intestine are shown in Figure 6. Two hours after oral administration, the labeled strain was detected in all intestinal contents, with a positive rate of over 60%. In the small intestine (duodenum, jejunum, and ileum), the concentration of the RS-09 bacteria decreased over time, reaching the lowest value at 48 h, with positive rate 3.98, 17.0, and 27.3% relatively. However, in the large intestine (cecum, colon and rectum), the fluorescence intensity first increased and then decreased, reaching the lowest value 48 h, with positive rate 42.33, 35.63, and 34.8% relatively. On 96 h, *L. plantarum* RS-09 was predominantly localized in the cecum, colon and rectum, with positive rate 69.4, 71.9, and 74.2% relatively.

**FIGURE 7:**
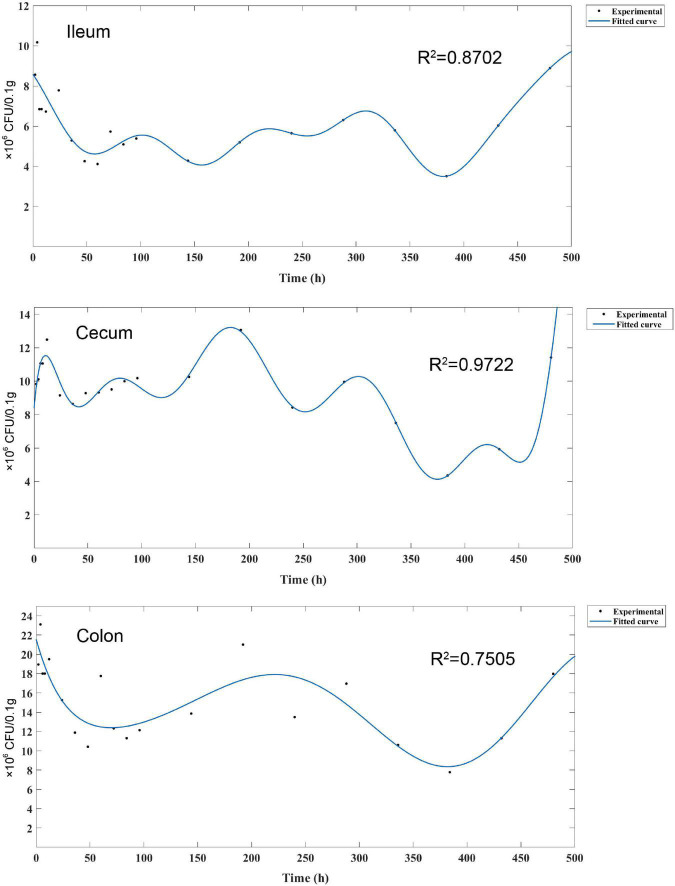
Fourier series model for experimental measured data. Fourier model of *L. plantarum* RS-09 dynamics show high harmonics. The measured Fourier transform is the average over Fourier transforms of bacterial count levels that were measured three times.

To further analyze the persistence process and pattern of *L. plantarum* RS-09 in the intestinal tract, we monitored the strains for 20 days and established a kinetic model. FCM results showed changes of *L. plantarum* RS-09 persistence in various segments of the intestine. Figure 7 illustrates a 480-h curve about the bacterial intensity and distribution in the intestine. The curve was fitted using Fourier Series Models by Matlab. Fluorescence value correlated with the number of viable bacteria (CFU/0.1 g) isolated from different parts of the intestine at corresponding time points. The strongest correlation was calculated for cecum (R^2^ = 0.9722) and ileum (R^2^ = 0.8702), but lower for colon (R^2^ = 0.7505). Fourier model of *L. plantarum* RS-09 dynamics show that an obvious decrease on the number of RS-09 was found at 48 h in the intestine, and the change in bacterial concentration increased after 48 h. The fluorescent signal of *L. plantarum* RS-09 in cecum displayed levels similar to those observed in colon and ileum from day 14 until 20. After 20 days, RS-09 could colonize in the intestinal tract steadily.

### Monitoring *L. plantarum* RS-09 in the Gastrointestinal Tract of Mouse

**FIGURE 8:**
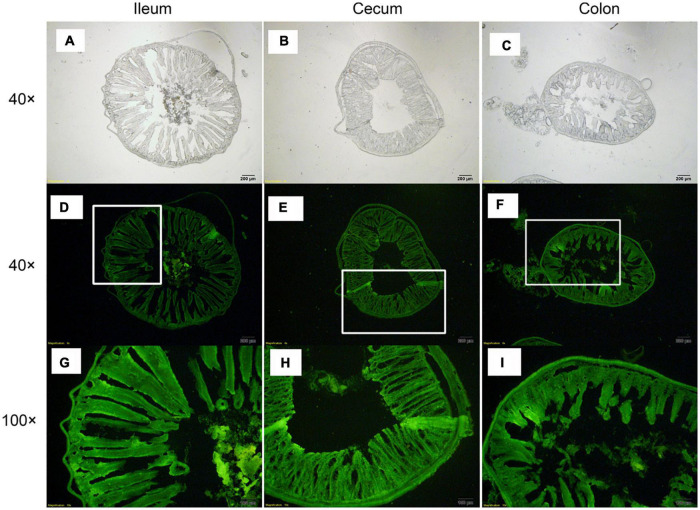
Distribution of *L. plantarum* RS-09 in frozen sections of intestinal tissue. **(A,D,G)** *L. plantarum* RS-09 in the ileum was assessed; **(B,E,H)** *L. plantarum* RS-09 in the cecum was assessed; **(C,F,I)** *L. plantarum* RS-09 in the colon was assessed. Scale bars, 200 μm (top; original magnification, ×40) and 100 μm (bottom; original magnification, ×100).

At 96 h after oral administration, a fluorescence microscope was used to observe CFDA/SE-labeled *L. plantarum* RS-09 in the ileum, cecum, and colon, which are the sites with long persistence. The results showed that the fluorescently-labeled strain could be observed in the intestinal mucosa and submucosa (Figure 8). In the ileum, cecum and colon, the fluorescently-labeled strain was presented in the intestinal mucus and lumen and closely attached to the epithelial cells. In addition, the labeled bacteria were observed in the lamina propria and muscularis mucosae. These results were consistent with those of FCM.

## Discussion

In this study, we created a novel method for real-time quantitative analysis of the adhesion process of probiotics in the gastrointestinal tract. By using CFDA/SE-labeled *L. plantarum* RS-09, we analyzed the spatiotemporal persistence patterns of *L. plantarum* in the gastrointestinal tract to establish a kinetics model of bacterial strain growth. These studies provide new insights into the long-term persistence of *L. plantarum* in the gastrointestinal tract.

Probiotic strains tolerance to acidic environment and bile salts are two criteria for a candidate probiotic strain to survive the GIT (Nami et al., 2019). The strain of *L. plantarum* described in this study was isolated from fermented apple juice and appears to have strong resistance to acid and bile salts. Lactic acid bacteria isolated from fermented apple juice have been adapted to the harsh fermentation conditions and, therefore, it is easier for them to survive, adhere to, and colonize the gastrointestinal tract compared to other strains. Many studies have shown that the isolated lactic acid bacteria from plant fermentation products have strong probiotic functions (Caggia et al., 2015; Ferrari Ida et al., 2016). In addition, bile tolerance can contribute to the ability of lactobacilli to persist in the gastrointestinal tract (Spivey et al., 2014). Previous reports considered *Lc. mesenteroides* as a bad probiotic candidate because of its low colonization rates of the large intestine, probably due to poor tolerance to acid and bile salt (Barache et al., 2020). In our study, RS-09 showed acid tolerance when grown at pH 2.5 or 3.5 and did not encounter growth reduction in the presence of 0.3% bile salts. This augments the potential capability of the strain to survive similar harsh conditions found in the GIT.

To date, the methods for the determination of bacterial adherence, including microscopic counting, double-antibody labeling and enzyme activity detection, all have disadvantages (Falk et al., 1994). For instance, microscopic counting is a cumbersome and subjective method to quantify adherent bacteria. FCM with double-antibody labeling to detect bacterial adherence is susceptible to non-specific binding of primary and secondary antibodies, which affects the ability to accurately determine adherence. The FITC method has been used to label microorganisms for quantitative analysis, but the fluorescence intensity decreased within 6–12 h and the signal gradually faded with the growth of the bacteria because of the short half-life of FITC in living systems (Kasugai et al., 2000). This is not conducive to long-term observations. In our study, CFDA/SE was used to label *L. plantarum* RS-09. This is a method modified from the study by Hodgkin et al. (1996), in which they used CFDA/SE to label lymphocytes for detecting lymphocyte proliferation/differentiation or for *in vivo* tracking. Among fluorescent dyes, CFDA/SE dyes have many advantages such as strong fluorescence, low toxicity and good durability. In addition, FCM detection of CFDA/SE-labeled strains can quantify both the ratio of bacteria to cells and the number of bacteria adhering to each cell. The results of the *L. plantarum* RS-09 adherence rate can be analyzed by fluorescence microscopy and FCM. Our results indicate that using CFDA/SE to label RS-09 with the use of a fluorescence-activated cell sorter (FACS) to analyze the adherence of strains to epithelial cells is a sensitive and durable technique and provides a more stable fluorescent labeling model.

The tolerance to the gastrointestinal environment of *Lactobacillus* species is related with its adhesion to intestinal epithelial cells. Based on scanning electron microscope (SEM) analysis, the morphology of RS-09 became short under bile salt stress. This result was consistent with another study that *L. plantarum* ATCC14917 cells was long rods in the acid and control groups, but short rods in the alkali group (Wang et al., 2018). HT-29 cells are ideal models for studying bacterial adherence *in vitro* and the RS-09 strain was highly adherent to these cells. The results showed that the adherence of the RS-09 strain to the HT-29 cells was minimally affected by the pH of the testing conditions. This lays the foundation for the persistence of this strain in the gastrointestinal tract.

Studies have shown that the survival rate of bacteria in the gastrointestinal tract is crucial for adherence. Fewer bacteria are generally present in the duodenum because of the low pH in the stomach and the fluidity of the intestinal contents (Breeuwer et al., 1996). However, the persistence rate of *L. plantarum* RS-09 in the duodenum at 96 h was 12.8% in this study. Van Zyl et al. (2018) also revealed the presence of viable cells of *L. plantarum* 423 in the small intestine at 4 and 24 h post gavage by the CFU counts, but no bioluminescent signals could be detected. This might be caused by reduced cell activity or the inefficient bioluminescence emission during passage in the stomach and duodenum (Van Zyl et al., 2018). This indicates that the RS-09 strain has a strong resistance to acid and bile salts, allowing this strain to have a probiotic effect further in the intestinal tract after surviving the gastric acid environment. Meanwhile it implies that the fluorescent signal of CFDA/SE is stable under the low pH gastrointestinal environment. At 96 h, fluorescent cells of RS-09 were predominantly localized in the cecum and colon, which are consistent with *L. plantarum* 423.

Although we can use HT-29 cells to evaluate the *in vitro* performance of *L. plantarum*, the comments of Greene and Klaenhammer that an *in vitro* cell model has limitations in its ability to reflect *in vivo* features are true (Hertel et al., 1993). In previous studies, the adhesion and persistence of gastrointestinal probiotics in the body were analyzed by collecting stool samples and performing plate counting to determine the number of colonizing bacteria. However, this method does not truly reflect the persistence in the intestine, and it is impossible to analyze the regional and temporal characteristics of probiotic persistence. In this study, a CFDA/SE-labeled strain administered to mice was used to quantify the strain in the intestinal tract *via* a real-time analysis of the intestinal contents by FCM. Earlier reports also have shown that FITC can label the bacterial strain and monitor the distribution of *Lactobacillus kefiranofaciens* ZW3 in the ileum and colon by detecting contents (Xing et al., 2018). FITC fluorescence imaging measured only the labeled cells, but not the overall level of colonization. While CFDA-SE is an effective and popular dye to monitor cells division. When a CFDA-SE -labeled cell divides, its progeny is endowed with half the number of carboxyfluorescein-tagged molecules.

Our study showed that the cecum is the segment with highest number of viable bacteria over time. The murine cecum may be the site where microorganisms adapt to the gastrointestinal environment and where the activation of genes required for colonization of the colon occur (Cronin et al., 2008). In addition, during long-term monitoring, we noticed that the fluorescence intensity of the RS-09 strain was lowest in all segments of the intestine 48 h after administration, after which it rebounded. This finding is consistent with that of the study by Han et al. (2015), in which the FCM detection rate of GFP-tagged *L. plantarum* reached the lowest value in the duodenum and ileum of goats after 42 h and then started to increase by 72 h. The specific mechanism is still unclear. After oral administration of high-dose probiotics, it may take 48 h for the bacteria to adapt to the gastrointestinal environment. In addition, the persistence patterns of *L. plantarum* RS-09 in the small intestine (duodenum, jejunum and ileum) and the large intestine (cecum, colon and rectum) were different. This result was consistent with another study that treated mice with the probiotic *Lacticaseibacillus rhamnosus*, where the number of colonization strains increased from the proximal to the distal small intestine (duodenum < jejunum < ileum) (Li et al., 2019). The persistence rate of *L. plantarum* in the large intestine was higher than that in the small intestine. This may be related to the structure of the intestine. The small intestine is slender, long, and impacted by bile, pancreatic juices, and various digestive enzymes. Thus, the persistence rate of probiotics is lower in the small intestine than in the large intestine.

Tissue sections were observed under a microscope to assess the specific contact sites between the bacteria, the submucosal cells, and cells from the muscularis mucosae (Vinderola et al., 2004). Considering the complex microbial environment in the stomach and intestines, we are unable to fully understand the fate of lactobacilli. Also, CFDA-SE-stain is suitable for cryosectioning applications and can withstand the processing with snap freezing and sectioning (Lönnqvist et al., 2019). Therefore, the fluorescence microscopy findings in the frozen sections will help us to understand the specific persistence sites and modes of action of microorganisms *in vivo*. Overall, the gastrointestinal persistence of *L. plantarum* RS-09 compared well to several other commercial probiotic strains, including *L. plantarum* 299v, *L. gasseri SBT2055* and *L. rhamnosus* GG.

## Conclusion

This study was carried out mainly to elucidate the *in vitro* and *in vivo* patterns of adherence and persistence of *L. plantarum* RS-09. We used, for the first time, bacterial CFDA/SE labeling technology to analyze the persistence of probiotics in a long- term *in vivo* tracking experiment. This model can be used to monitor the survival of strains *in vivo* and to further study the interactions between microorganisms and hosts by means of this FCM technique.

## Data Availability Statement

The original contributions presented in the study are included in the article/supplementary material, further inquiries can be directed to the corresponding author/s.

## Ethics Statement

The animal study was reviewed and approved by the Laboratory Animal Welfare and Ethics Committee of Ludong University.

## Author Contributions

XyZ and CZ designed the experiments. LY, JZ, XY, and GC carried out the experiments. HZ, WT, and YL analyzed the experimental results. MW and HJ wrote the manuscript. LJ and XxZ reviewed and edited the manuscript. All authors contributed to the article and approved the submitted version.

## Conflict of Interest

The authors declare that the research was conducted in the absence of any commercial or financial relationships that could be construed as a potential conflict of interest.

## Publisher’s Note

All claims expressed in this article are solely those of the authors and do not necessarily represent those of their affiliated organizations, or those of the publisher, the editors and the reviewers. Any product that may be evaluated in this article, or claim that may be made by its manufacturer, is not guaranteed or endorsed by the publisher.
